# An Approach to Estimating State‐Level Medicaid Nursing Home Spending

**DOI:** 10.1111/1475-6773.70112

**Published:** 2026-04-05

**Authors:** Hannah C. Ratliff, Kierstdea R. Petzold, Donovan T. Maust, Kali S. Thomas

**Affiliations:** ^1^ University of Michigan Medical School Ann Arbor Michigan USA; ^2^ Center for Clinical Management Research VA Ann Arbor Healthcare System Ann Arbor Michigan USA; ^3^ Johns Hopkins School of Nursing Baltimore Maryland USA

**Keywords:** health policy, healthcare access, LTCFocus, LTSS expenditures, Medicaid, payment systems, policy evaluation, validation

## Abstract

**Objective:**

To create and validate a measure of state‐level, average daily Medicaid nursing home spending rates using publicly available data sources.

**Study Design:**

We created a new state‐level measure of average daily Medicaid nursing home payment rates, calculating Medicaid spending per day divided by estimated Medicaid days. We compared this new measure to existing benchmarks in 2004 and 2019 and estimated correlations (Pearson and Spearman rank coefficients).

**Data Sources and Analytic Sample:**

To create the new measure, we used data from Brown University's Long‐Term Care Focus website and the Medicaid Long‐Term Services and Supports Annual Expenditure Report, covering nursing home spending and characteristics from most states.

**Principle Findings:**

The measure of state‐level average daily Medicaid nursing home spending demonstrated strong positive correlations with both historical data (Pearson [*r*] = 0.70, Spearman rank [ρ] = 0.80) and government‐published estimates (*r* = 0.68, ρ = 0.63).

**Conclusions:**

The new, validated measure offers a reliable and transparent method for comparing Medicaid nursing home payment rates across states using only publicly available data. Importantly, this approach enables timely, cross‐state comparisons without the need for restricted or proprietary data, improving transparency and removing barriers to policy evaluation.

## Summary

- What is known about this topic
  ○ Medicaid is a primary payer for nursing home services in the United States, with state budgets significantly impacted by nursing home spending.
  ○ Currently, there is no reliable, standardized measure of state‐level, average Medicaid nursing home payment rates, making it hard to monitor trends or evaluate policies.
- What this study found
  ○ We created a new measure of state‐level, average daily Medicaid nursing home payment rates using publicly available data.
  ○ This proposed new measure demonstrated strong, positive correlations with both historical data from LTCFocus and a recent government‐published estimate.
  ○ This method enables timely, cross‐state comparisons of Medicaid nursing home payment rates without relying on restricted or proprietary data, improving transparency and supporting policy evaluation.

## 1 Introduction

Given the aging of the United States (U.S.) population, long‐term services and supports (LTSS) are a critical component of the nation's healthcare system. LTSS are a range of care services typically provided to older adults who require assistance with healthcare (e.g., medication management, driving to appointments) or personal care activities (e.g., eating, bathing, walking). These services are available at home, in community settings, or in facilities. Nursing homes, which serve approximately 1.3 million individuals every year [1], are one such facility that provides these services [2]. Annually, long‐term care accounts for 36% of Medicaid spending, and more than half of all nursing home residents in the U.S. rely on Medicaid to pay for their care [3, 4]. Since each state administers its own Medicaid program with federal support, states may vary widely in their levels of funding for nursing home care, with significant implications for state budgets but also for the care that those states' nursing homes can deliver to residents. Moreover, restrictions or expansions in Medicaid eligibility or reimbursement rates can impact access to and quality of care. For example, increasing Medicaid payments has been shown to improve the likelihood that nursing homes meet quality benchmarks, such as effective pain management, prevention of pressure ulcers, and maintenance of residents' functional status [5]. Quantifying Medicaid nursing home spending is necessary to inform public health strategy and policy decisions about LTSS in the U.S.

Despite the importance of this information, there are few publicly available sources that quantify state‐level average Medicaid nursing home payment rates. Long‐Term Care Focus (LTCFocus), a collaborative based at Brown University and supported in part by the National Institute on Aging, provides one historical exception, offering state‐level average daily Medicaid nursing home payment rates for the years 2000 to 2009 [6]. These data were collected from a survey sent to state Medicaid administrators; the specific survey item asked states to report their average daily nursing home reimbursement rate. A more recent estimate from 2019 is available in a report by the Medicaid and CHIP Payment and Access Commission (MACPAC) [7]. MACPAC's estimates were calculated from allowed, fee‐for‐service and managed care payments using beneficiary‐level data from the Transformed Medicaid Statistical Information System (T‐MSIS). Allowed payments are the actual amounts Medicaid reimburses for services, which can differ from the billed amounts submitted by nursing homes. Given the critical role of Medicaid funding for nursing homes, as well as significant differences in spending among states, an approach to calculate current estimates of state‐level Medicaid nursing home payment rates is needed. This is particularly important following passage of the One Big Beautiful Bill Act of 2025, which enacted funding cuts and policy changes to Medicaid, including restrictions on how states can finance their Medicaid programs [8]. Without timely, consistent, and comparable metrics, it will be challenging to monitor the downstream effects of such policy changes across states on nursing homes. Therefore, the objective of this study was to create a measure of state‐level, Medicaid nursing home spending rates, and to validate this measure with historical data from LTCFocus and a recent government‐published estimate.

## 2 Methods

### 2.1 Data Source and Sample

To calculate our new estimate of Medicaid spending per nursing home resident‐day, we used the Medicaid LTSS Annual Expenditure Reports released by the Centers for Medicare & Medicaid Services (CMS) and Brown University's LTCFocus [6, 9]. The Medicaid LTSS annual expenditure data released by CMS is reported by federal fiscal year (October 1–September 30) and includes state‐level nursing home Medicaid spending calculated using Medicaid CMS‐64 Financial Management Report (FMR) net services data and state‐reported managed LTSS expenditures. The CMS‐64 provides detailed state spending by service category (e.g., nursing home) including fee‐for‐service base payments (from line 3A) and supplemental payments (from line 3B). Managed LTSS data were collected directly from states with active managed LTSS programs since CMS‐64 does not distinguish managed LTSS spending from other Medicaid managed care spending. We used LTCFocus data to obtain state‐level nursing home information.

### 2.2 Proposed New Measure

Our aim was to calculate the total Medicaid nursing home spending in each state divided by the total number of Medicaid days in nursing homes in that state:

(1) $$\frac{\text{LTSS Nursing Home Spending}/365\;}{\text{Average Medicaid paid days in nursing homes}}$$

The numerator for our proposed new measure came from the CMS Medicaid LTSS Annual Expenditure Report for the year of interest. Using LTCFocus data, we calculated a state‐specific denominator of average Medicaid days in nursing homes using three specific variables for each state, multiplying: (total number of nursing home beds in each state [*totalbeds_sta*]) by (the occupancy rate [*occpct_sta*]) by (proportion of Medicaid‐supported residents [*paymcaid_sta*]):

(2) $$\begin{array}{c} \text{Average Medicaid paid days in nursing home}\\ =\text{number of nursing home beds}*\text{occupancy rate}\\ {}*\text{proportion supported}\;\mathrm{by}\;\text{Medicaid} \end{array}$$

Total nursing home beds come from the annual certification survey (OSCAR/CASPER), the occupancy rate is the proportion of nursing home beds occupied on the date of the annual survey, and the proportion of Medicaid‐supported residents is the proportion of residents whose primary support is Medicaid on the date of the annual survey.

### 2.3 Analyses

We used our proposed new measure to calculate estimates for 2004 and 2019 and validated them against previously reported estimates from those same years. The 2004 estimate comes from LTCFocus data and represents the state‐level average daily Medicaid nursing home payment rates for the years 2000 to 2009, adjusting to 2004 dollars (LTCFocus variable name *adj_mrate_sta*). Since all values were adjusted to 2004 dollars, we selected 2004 for our validation year. LTCFocus did not report estimates for Hawaii and Alaska in 2004, so we excluded them from our 2004 validation. For a second and more recent external validation, we calculated state‐level estimates for 2019 and compared them to estimates from the 2019 MACPAC report [7]. The 2019 estimates represent state‐level average Medicaid nursing home base payment rates and exclude supplemental Medicaid payments. MACPAC does not include estimates for Idaho, New Hampshire, and Alaska, so we excluded these states from our 2019 validation.

We compared our proposed new measure first to the 2004 estimates from LTCFocus and then to the 2019 estimates from MACPAC. We then compared the sets of 2004 and 2019 estimates by calculating the differences between the measures (e.g., proposed new measure estimates—2004 estimates) and the corresponding absolute differences. To assess validity, we used Loess plots to visualize the relationship between the estimates from our proposed new measure and those estimates that were previously reported in 2004 and 2019; a straight line in the plot indicates a linear relationship between the estimates from our proposed new measure and the previously reported estimates, suggesting agreement between the two measures. We also examined the strength of these relationships using Spearman rank correlation coefficients for all comparisons. Pearson correlation coefficients were calculated if the Loess plots indicated that the association between estimates was approximately linear. Correlation values from 0.60 to 0.79 were categorized as moderately strong, values above 0.80 were considered very strong, and values of 1.0 were considered perfect [10]. Lastly, we identified potential outliers, defined as values lying more than 1.5 times the interquartile range (IQR) below the first quartile or above the third quartile, and conducted a sensitivity analysis by reexamining the strength of the correlations after removing these outliers.

## 3 Results

**FIGURE 1:**
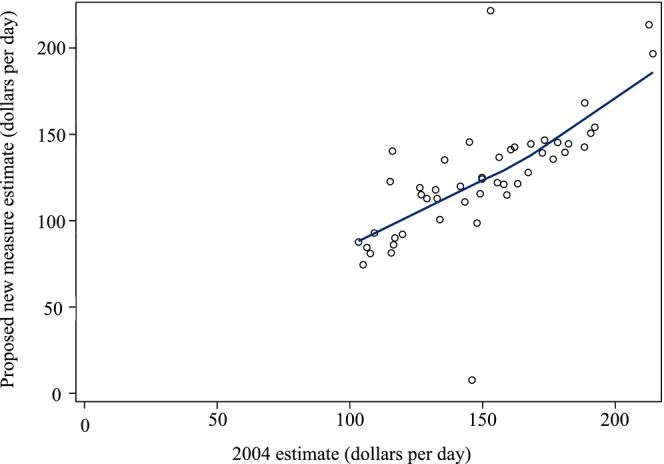
Loess plot of Medicaid payment rate per day for 2004 estimate vs. proposed new measure estimate, 2004. *N* = 48. Alaska and Hawaii were excluded from the 2004 estimate, reported by LTCFocus. Values are reported in 2004 dollars.

Using data from 2004, our proposed new measure estimated a state‐level median average nursing home Medicaid rate of $122.30 per day (interquartile range [IQR]: [$105.73, $142.60]) compared to the 2004 estimate of $149.44 per day (IQR: [$126.64, $170.37]; Table 1). The median difference was ‐$26.78 per day (IQR: $16.95, $35.64), and the absolute difference was $26.91 per day (IQR: $19.50, $37.46). The Loess plot displayed a linear relationship between the two estimates (Figure 1). The Pearson correlation coefficient was 0.70, indicating a moderate association, and the Spearman rank correlation was 0.80, indicating a strong association.

**TABLE 1:** Comparing the proposed new measure estimate to previously reported estimates in 2004 and 2019.

	Proposed new measure estimate versus 2004 estimate, 2004 (*n* = 48)^a^	Proposed new measure estimate versus 2019 estimate, 2019 (*n* = 47)^b^
	Median (IQR), $	
Comparator estimate^c^	149.44 (126.64, 170.37)	201.73 (185.85, 227.42)
Proposed new measure estimate: average daily nursing home Medicaid rate	122.30 (105.73, 142.60)	194.02 (159.22, 233.13)
Differences (proposed new measure estimate‐comparator estimate)	26.78 (16.95, 35.64)	18.14 (−7.86, 33.66)
Absolute value of differences	26.91 (19.50, 37.46)	30.91 (15.27, 46.99)
Correlation measure	Coefficient	
Pearson (*r*)	0.70	0.68
Spearman rank (ρ)	0.80	0.63

*Note:* 2004 estimates are in 2004 dollars; 2019 estimates are in 2019 dollars.

^a^ Excludes Alaska and Hawaii.

^b^ Excludes Alaska, New Hampshire and Idaho.

^c^ 2004 estimate reports average daily nursing home Medicaid rate; 2019 estimate reports nursing home Medicaid base payment.

Arizona and Pennsylvania were outliers in 2004 with absolute differences between the proposed new measure estimates and 2004 estimates of $138.39 and $68.58 per day, respectively. A sensitivity analysis removing Arizona and Pennsylvania from the comparison resulted in a Pearson correlation of 0.87 and a Spearman rank correlation of 0.85, both now indicating a strong association between the two estimates (see Appendix S1).

**FIGURE 2:**
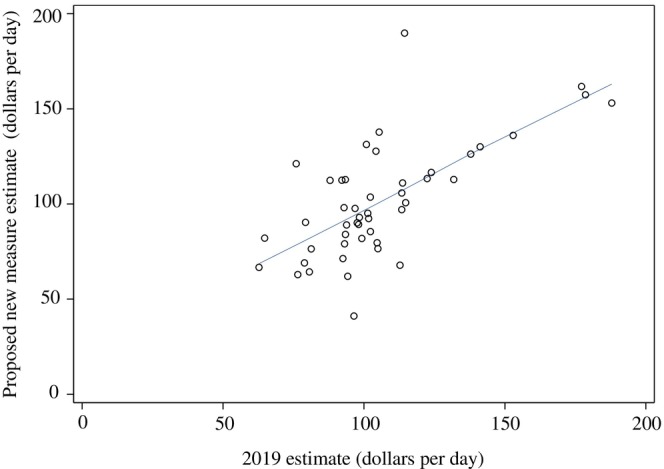
Loess plot of Medicaid payment rate per day for 2019 estimate versus proposed new measure estimate, 2019. *N* = 47. Alaska was excluded from LTCFocus due to small sample size, and thus excluded from the proposed new measure estimate; Idaho and New Hampshire were excluded from the 2019 estimate, reported by MACPAC, due to missing payment data. Values are reported in 2019 dollars.

Using data from 2019, our proposed new measure showed a state‐level median average nursing home Medicaid rate of $194.02 per day [IQR $159.22, $233.13], while the 2019 estimates reported a median Medicaid base payment of $201.73 (IQR [$185.85, $227.42]; Table 1). The difference was $18.14 per day (IQR: [−$7.86, $33.66]), with an absolute difference of $30.91 per day (IQR: [$15.27, $46.99]). The Loess plot (Figure 2) showed a linear relationship between the two estimates. Pearson and Spearman rank correlation coefficients were 0.68 and 0.63, respectively, indicating a moderate association between the two measures.

Illinois and New York were outliers in 2019 with absolute differences between the proposed new measure estimates and 2019 estimates of $110.68 and $150.81, respectively. A sensitivity analysis removing Illinois and New York from the comparison resulted in a Pearson correlation of 0.76, indicating a strong relationship, and a Spearman rank correlation of 0.62, indicating a moderate relationship between the two estimates (see Appendix S1).

## 4 Discussion

Our study presents a simple method for estimating state‐level average daily Medicaid nursing home spending rates by combining information from the Medicaid Long‐Term Services and Supports (LTSS) Annual Expenditure Reports published by CMS with publicly available LTCFocus data. The strong correlations between our proposed new measure and a historical estimate from LTCFocus and a single year estimate from MACPAC support its validity, even when state outliers are included. Importantly, this new approach facilitates timely cross‐state comparisons of Medicaid payment rates without the need for restricted or proprietary data thereby improving transparency and removing barriers to policy evaluation.

Our study has several limitations. The LTSS estimate used in our proposed new measure relies in part on managed care payments, some of which were missing or reported as aggregate managed care payments that had to be estimated to the nursing home setting [11]. Aggregate reports may be related to how managed care programs are structured. For example, in 2004, Arizona's Medicaid program operated through an integrated managed care model that combined funding for both acute and long‐term care services; this made direct reporting of nursing home managed care spending challenging [12], and possibly led to an underestimation of Arizona's Medicaid nursing home spending rates that year. Since 2004, Medicaid nursing home payments have continued to shift toward managed care, making this limitation more problematic for our 2019 validation. Indeed, the 2019 LTSS expenditure report included extensive notes about potential reporting inconsistencies across a number of states. The lower correlation in 2019 compared to 2004 likely resulted from increased prevalence and complexity of reporting of managed care along with differences in how the two estimates were calculated. The 2019 estimate used to validate our method relied on beneficiary‐level data, while our proposed measure relies on aggregate data, which may introduce errors in the estimates. Following 2019, LTSS estimates use beneficiary‐level data, which should improve validity but warrants longitudinal validation. We were also unable to reconcile all differences in inflation adjustment methods used across different data sources. To support comparisons, we used data for our proposed new measure from years to which the 2004 estimate and 2019 estimate had already been adjusted. Lastly, our proposed new measure represents the estimated average payment at the state level and does not capture inter‐state variation in how these payments are made (e.g., retrospective fee‐for‐service, prospective payment, case‐mix adjusted, managed care) or facility variation in actual payment. Despite these limitations, our method provides an approach applicable across many states and years and its reliance on publicly available data makes it replicable and accessible for researchers. We encourage researchers to carefully review accompanying data notes and state‐level differences when considering the use of our proposed new measure.

In summary, this method for measuring state‐level average Medicaid nursing home payment rates provides a valuable tool for research and policy analysis. Having a reliable and comparable estimate of Medicaid nursing home payment rates supports future studies examining how changes in Medicaid spending impact the availability, quality, and organization of long‐term care for various populations. This can aid future researchers in studies that inform future policy reforms aimed at improving nursing home care.

## Funding

This work was supported by the National Institute on Aging, RF1AG08201.

## Conflicts of Interest

The authors declare no conflicts of interest.

## Supporting information


**Data S1:** Sensitivity analyses.

## Data Availability

The data that support the findings of this study are openly available in LTCFocus at https://ltcfocus.org, reference number https://doi.org/10.26300/h9a2‐2c26 and Medicaid.gov at https://www.medicaid.gov/medicaid/long‐term‐services‐supports/reports‐evaluations.
