# Supplementary material for: An Approach to Estimating State‐Level Medicaid Nursing Home Spending
Source: Health Serv Res. 2026 Apr 5;61(2):e70112. doi: 10.1111/1475-6773.70112 (PMC13052202; doi:10.1111/1475-6773.70112)
Supplement: Supplementary file 1 — Data S1: Sensitivity analyses. [file HESR-61-0-s001.docx]

**Appendix**

**Supplement 1.** Sensitivity analyses.

|  | Proposed new measure estimate vs. 2004 estimate, 2004  (n = 46)^a^ | Proposed new measure estimate vs. 2019 estimate, 2019  (n = 45)^b^ |
| --- | --- | --- |
|  | **Median (IQR), $** | |
| Comparator measure^c^ | 149.44 (126.37, 172.54) | 201.73 (185.85, 226.83) |
| Proposed new measure estimate: average daily nursing home Medicaid rate | 122.30 (110.84, 142.57) | 194.02 (163.73, 236.68) |
| Differences (proposed new measure estimate-comparator estimate) | 26.78 (17.49, 34.29 ) | 18.14 (-2.69, 32.77) |
| Absolute value of differences | 26.78 (19.49, 34.29) | 28.23 (15.27, 42.40) |
| **Correlation measure** | **Coefficient** | |
| Pearson (*r*) | 0.87 | 0.76 |
| Spearman rank (ρ) | 0.85 | 0.62 |

*Note:* LTCFocus values are in 2004 dollars; MACPAC values are in 2019 dollars.

^a^Excludes Alaska, Hawaii, Pennsylvania, and Arizona.

^b^Excludes Alaska, New Hampshire, Idaho, New York, and Illinois.

^c^2004 estimate reports average daily nursing home Medicaid rate; 2019 estimate reports nursing home Medicaid base payment.
